# Stable optical trapping and sensitive characterization of nanostructures using standing-wave Raman tweezers

**DOI:** 10.1038/srep42930

**Published:** 2017-02-17

**Authors:** Mu-ying Wu, Dong-xiong Ling, Lin Ling, William Li, Yong-qing Li

**Affiliations:** 1 School of Electronic Engineering, Dongguan University of Technology, Dongguan, Guangdong, P.R. China; 2 Department of Physics, East Carolina University, Greenville, North Carolina 27858-4353, USA

## Abstract

Optical manipulation and label-free characterization of nanoscale structures open up new possibilities for assembly and control of nanodevices and biomolecules. Optical tweezers integrated with Raman spectroscopy allows analyzing a single trapped particle, but is generally less effective for individual nanoparticles. The main challenge is the weak gradient force on nanoparticles that is insufficient to overcome the destabilizing effect of scattering force and Brownian motion. Here, we present standing-wave Raman tweezers for stable trapping and sensitive characterization of single isolated nanostructures with a low laser power by combining a standing-wave optical trap with confocal Raman spectroscopy. This scheme has stronger intensity gradients and balanced scattering forces, and thus can be used to analyze many nanoparticles that cannot be measured with single-beam Raman tweezers, including individual single-walled carbon nanotubes (SWCNT), graphene flakes, biological particles, SERS-active metal nanoparticles, and high-refractive semiconductor nanoparticles. This would enable sorting and characterization of specific SWCNTs and other nanoparticles based on their increased Raman fingerprints.

Optical trapping integrated with spectroscopic techniques is attractive for manipulation and characterization of nanoscale particles^1^,^2^,^3^. Optical tweezers based on single-beam gradient force have become a powerful tool for manipulating micrometer-sized objects including biological particles^4^,^5^,^6^ and have been applied for single-molecule force spectroscopy^7^,^8^ and photonic force microscopy^9^. The integration with Raman spectroscopy, called Raman tweezers, allows chemical and physical analysis of single optically trapped particles via molecular vibrational fingerprints^10^,^11^,^12^. Raman tweezers have been used to monitor molecular dynamics of single living cells^13^,^14^, identify microorganisms^15^, and analyze polystyrene nanoparticles^16^ and metal colloids for surface-enhanced Raman spectroscopy (SERS)^17^,^18^. However, the application of optical tweezers for stable trapping of nano-sized objects in the 10–100 nm range is not straightforward but remains a great challenge because of the weak gradient force^1^,^19^, although Raman tweezers were reported for analysis and manipulation of single-walled carbon nanotubes (SWCNTs)^20^,^21^, graphene flakes^22^,^23^, and SERS-active metal nanoparticles^24^,^25^,^26^. To stably confine a particle, the gradient force must be greater than the forward scattering force and overcomes Brownian motion of the particle in liquid^4^. However, the gradient force acting on a nanoparticle decreases significantly as the third power of the particle’s size and thus a high optical power is required to confine nanoparticles against the destabilizing effects of Brownian motion^1^,^4^. With a moderate laser power, the escape of nanoparticles from the optical trap prevents effective spectroscopic analysis and unstable trapping lowers Raman signals of the trapped nanoparticles.

A specific interest is optical sorting and characterization of SWCNTs^20^,^21^, because SWCNTs show remarkable chirality-dependent properties. The production of SWCNTs often produces random chiralities while some SWCNT chiralities are metallic and the others are semiconductors^21^. Although selective trapping and aggregation of SWCNTs with specific chiralities was observed with single-beam Raman tweezers^20^,^21^, stable trapping and spectroscopic identification of an individual SWCNT for a prolonged period was not achieved^21^. Recently, several approaches were developed to trap and manipulate nanoparticles that would prompt spectroscopic characterization. An increase in trapping lifetime for optically trapped 100-nm gold particles and 350-nm silica particles was demonstrated using feedback control of position and intensity^19^. Carbon nanotubes, graphene flakes, and semiconductor nanowires were shown to be trapped by a single focused laser beam due to their highly anisotropic geometries^1^,^20^,^21^,^22^,^27^. Metal nanoparticles were trapped by the plasmon-enhanced gradient forces^28^,^29^,^30^ and plasmon nano-optical tweezers were developed to trap nanoparticles by the field enhancement in subwavelength scale^31^,^32^,^33^. In this paper, we present a standing-wave Raman tweezers for stable trapping and sensitive characterization of individual isolated nanostructures in an aqueous solution by combining confocal Raman spectroscopy and standing-wave optical traps. The standing-wave Raman tweezers has stronger intensity gradients for nano-sized particles, eliminates axial scattering forces, and increases Raman scattering signals by a factor of 4–8 folds. We show that it enables prolonged trapping and analysis of an individual SWCNT with a specific chirality and single graphene flasks, and it allows to manipulate and analyze those nanoparticles that cannot be trapped by single-beam Raman tweezers (such as nanoparticles with high index of refraction, high absorption, or high reflection) with a low-power laser of a few mW at 780 nm.

## Materials and Methods

### Standing-wave Raman tweezers

**Figure 1:**
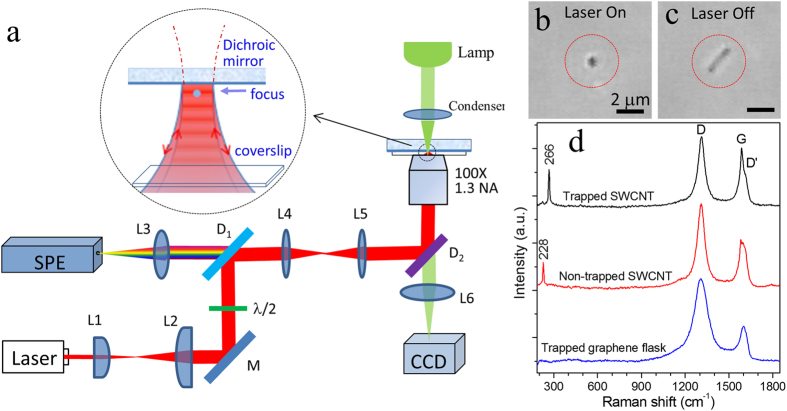
(**a**) Experimental setup of a standing-wave Raman tweezers (SWRT). The trapping laser is focused through an objective lens and reflected by a planar dichroic mirror to form a standing-wave optical trap in the sample chamber. Raman scattering from a trapped nanoparticle excited by the trapping laser is collected through the same lens and dichroic mirrors, focused into a spectrometer (SPE), and acquired with a CCD camera. A λ/2-waveplate is used to control the polarization of the trapping beam and M is a reflecting mirror. (**b**) The image of a trapped single-wall carbon nanotube (SWCNT) when the trapping laser is on (see Supplementary Movie 1). (**c**) The image of the SWCNT when the trapping laser is turned off and the SWCNT moves freely. (**d**) Raman spectra of a single optically trapped SWCNT, a SWCNT that is unable to trap, and a trapped graphene flake. The laser wavelength is 780 nm and the laser power is 5 mW with an acquisition time of 5 s.

The standing-wave Raman tweezers (SWRT) is the combination of a standing-wave optical trap (SWOT) and Raman spectroscopy. SWOT can be formed by the interference of a tightly focused Gaussian beam with the reflected beam by a dichroic mirror (Fig. 1a) or counter-propagating beams, which have previously been utilized to manipulate and transport dielectric or metal nanoparticles^34^,^35^,^36^. Comparing to single-beam Raman tweezers^10^,^11^,^12^, the SWRT have several advantages. First, in a SWOT the scattering force produced by the incident beam is balanced by the scattering force produced by the counter-propagating reflected beam. This property is particularly useful for trapping the highly refractive, reflective or absorbing nanoparticles (such as semiconductor, carbon nanotubes, and metal particles), which may not be trapped with single-beam OT^17^. Second, the SWOT has stronger axial intensity gradients and thus deeper potential wells for the same incident power, while the lateral focal waist is determined by the Abbe diffraction limit^37^. When an incoming Gaussian beam is reflected at the mirror, the intensity pattern of the standing wave near the mirror and the beam waist is given by I(z,  ψ)], where k = 2πn/λ is the wavenumber, n the index of refraction of the surrounding medium, λ the wavelength, I_0_ the intensity of the incident beam at the beam waist *w*_0_, and ψ the phase shift after the reflection by the mirror^37^. The distance between two nodes of the standing wave is given by λ/(2n) ≈ 0.3 μm (for λ = 780 nm and n = 1.33). Therefore, stable trapping of different kinds of nanoparticles is allowed in the SWOT with a low-power incident beam. Third, Raman scattering signal from an optically trapped nanoparticle in a SWOT is increased by a factor of 4–8 fold for the same incident power. If the particle’s size is much smaller than the wavelength (d ≪ λ/2n), Raman signal is increased by a factor of ~8-fold, because the intensity in the node position at which the nanoparticle is trapped is higher by 4-fold than that of the incident beam. The reflection of Raman scattering light by the dichroic mirror will increase the Raman collection efficiency by a factor of 2, since Raman scattering light emitted in backward direction is directly collected by the objective lens, whereas Raman scattering light emitted in forward direction will be reflected by the dichroic mirror and collected by the objective lens. If the particle size is comparable or larger than the wavelength (d > λ/2n), the average intensity across the particle is twice the intensity of the incident beam and thus the Raman signal is increased by a factor of ~4-fold. Zemánek *et al*. has calculated and compared the trap stiffness between the single-beam OT and the SWOT using a Gaussian beam and found that SWOT produces a much stronger axial trap stiffness (by a factor >10^6^) and about four times bigger radial trap stiffness than single-bean OT when the size of the particle is much smaller than the wavelength^3^^7^.

### Combination of a standing-wave optical trap and Raman spectroscopy

Figure 1a shows the experimental setup of a standing-wave Raman tweezers. A single laser beam at 780 nm from a semiconductor laser (TEC-300–0780–0500, Sacher Lasertecknik Inc.) coupled into a single-mode optical fiber is used both for Raman spectroscopy excitation and optical trapping. The laser source has a frequency bandwidth <1 MHz and a narrow band-pass filter (LL01–780–12.5, Semrock Inc.) is used to spectrally purify the laser beam. A pair of lens (L1 and L2) are used to expand the laser beam to fill the exit pupil of the objective with high numerical aperture (NA). A half-wave plate is used to change the polarization direction of the incident laser beam. The laser beam is introduced into the objective (100x, 1.3NA, Nikon) of an inverted microscope (TE-2000S, Nikon) through a dichroic mirror D_1_ (LPD02–785RU-25, Semrock Inc.), a pair of tube lens (L4 and L5), and a hot mirror D_2_ to form single-trap optical tweezers^12^.

The tightly focused laser beam is reflected by a planar dichroic mirror (>98% reflection for 750–1100 nm and >90% transmission for 400–700 nm) and interferes with the incident beam to form a SWOT in a sample chamber, which is made of a bottom glass coverslip and the reflecting dichroic mirror separated by a 50-μm spacer. When the focus of the objective is moved onto the reflecting surface or a distance <0.5 μm from it, the reflected beam is spatially matching the incident focused beam such that a stable SWOT is formed at the first node from the mirror, which can be sharply imaged by the microscope. Although the nanoparticles could be trapped in the first node, second node, or third node of the standing wave, only the nanoparticles trapped in the first node can be viewed clearly by the microscope. A slight adjust of the microscope focus can transport the trapped particle in the second node to the first node. On the other hand, when the focus of the objective is adjusted to a distance >10 μm below the reflecting surface, the reflected beam is too diverging to interfere with the incident focused beam such that the trapping is dominant by a single-beam optical tweezers. Therefore, by adjusting the focus distance of the objective, a standing-wave optical trap can be changed to a single-beam optical tweezers, and vice versa.

Raman scattering light from a trapped nanoparticle is collected with the same objective lens, passes through a dichroic mirror D1 and a long-pass filter (LP02–780RU-25, Semrock Inc.), and then focused onto the entrance slit of an imaging spectrograph (Triax 320, Horiba Scientific), which contains a CCD detector (PIXIS 100BR, Princeton Instruments) to record the Raman spectra of the trapped particles. In order to reject the out-of-focus light from the specimens, five pixels of the CCD detector are binned and the entrance slit of the spectrograph is set as 100 μm, corresponding to spatial filtering with a confocal pinhole. The bright-field or differential interference contrast (DIC) images of a trapped nanoparticle is illuminated with a lamp and recorded with a video CCD camera. The background spectrum is collected without a nanoparticle in the trap and subtracted from the Raman spectra of the trapped nanoparticles.

It should be noted that in the current experiment the SWOT is formed by the interference between the incident beam and the reflected beam from a reflecting mirror such that the optimum trapping position is very close to the mirror surface. In the further development, two counter-propagating tightly focused laser beams could be used to form a stable standing-wave optical trap for 3-dimensional manipulation of nanoparticles^38^.

## Results and Discussion

### Optical trapping and Raman spectroscopy of individual single-walled carbon nanotubes and graphene flakes

Short single-walled carbon nanotubes (SWCNTs, average diameter of 1.1 nm with a length of 1–3 μm), multi-walled carbon nanotubes (MWCNT, outside diameter of 50–80 nm and inside diameter of 5–15 nm with a length of 0.5–2 μm), and graphene flakes (1–5 μm in diameter) were obtained from US Research Nanomaterials Inc. and properly diluted in distilled water. The purity of SWCNTs was >90%. A drop of SWCNTs suspension (2 μL) was filled and sealed in the sample chamber. An individual SWCNT can be stably trapped in a SWOT and manipulated across the chamber well with an incident laser power of 5 mW (see Video1 in Supplementary Information). Figure 1b shows the image of a trapped isolated SWCNT in the first node of the SWOT. When the trapping laser was turned off, this SWCNT moved freely in water. Figure 1c shows the image of the moving SWCNT at an instant when the trapping laser is turned off, which indicates that this SWCNT has a length of ~2.1 μm. The image size of the trapped SWCNT in Fig. 1b shows that the SWCNT in the SWOT was forced to concentrate in the trap volume (~0.3 μm in lateral direction). Figure 1d shows Raman spectra of a single trapped SWCNT, a SWCNT that is unable to trap, and a single trapped graphene flake, respectively. The laser power for trapping was 5 mW with an acquisition time of 5 s. The Raman spectrum of the trapped SWCNT contains the G peak (~1581 cm^−1^), the D peak (~1310 cm^−1^), and the D′ peak (~1608 cm^−1^)^22^, as well as a strong radial breathing mode (RBM) (~266 cm^−1^)^20^,^21^. The G peak corresponds to the E_2g_ phonon at the Brillouin zone center, the D peak is due to the breathing modes of sp^2^ rings that requires a defect for its activation by double resonance, and the D′ peak is due to intravalley double resonance^22^,^23^. We assigned the presence of the large intensity of the D peak and D′ peak to the edges of the short length SWCNTs^22^. We did not exclude the possibility that the trapped object was a nanotube bundle which could be formed due to the unmatched pH value of the water dilution, although a trapped nanotube bundle likely generates more than one RBM modes or a broaden RBM mode.

Some individual SWCNTs cannot be stably trapped in the SWOT and they are likely pushed away from the trap or pushed upward and adhere on the surface of the dichroic mirror. The Raman spectrum of these non-trapped SWCNTs not only contains the G, D, and D′ peaks, but also contains very different RBM peaks (~228 cm^−1^ or 208 cm^−1^). The RBM frequency is strongly dependent on the chiralities of semiconducting or metallic tubes^21^. Typically, no RBM peaks were observed for single trapped graphene flakes (Fig. 1d). Previous studies showed an enhancement in trapping forces for semiconducting tubes by a near-infrared laser trap of 1064 nm wavelength^20^, but the enhancement for metallic tubes was found with a 633-nm laser trap^21^. In Fig. 1d, we noticed that the G peak (1500–1600 cm^−1^) of the trapped SWCNT consists of a sharp peak at ~1586 cm^−1^ with a shoulder at ~1570 cm^−1^, whereas in the non-trapped SWCNT the sharp peak at ~1586 cm^−1^ decreases significantly with a shoulder at ~1565 cm^−1^. It has been noted that the G peak of the SWCNTs splits in two superimposed components G^+^ (~1590 cm^−1^) and G^−^ (~1570 cm^−1^)^39^, corresponding to the modes with atomic displacement along the tube axis or along the circumferential direction, respectively. For metallic nanotubes, the G^-^ component is very broad in contrast to that of semiconducting nanotubes, which can be used to distinguish between metallic or semiconducting tubes^39^. We attributed the trapped nanotubes to the semiconducting group, based on the G-band spectra in Fig. 1d.

In previous studies with single-beam Raman tweezers^20^,^21^, a cloud of specific tubes that had high dipole polarizability but low absorption at the trapping wavelength were able to be trapped and characterized in the confocal volume of the optical trap due to the enhancement of the gradient force^21^. However, stable trapping of an individual SWCNT for a prolonged period was not observed in a single-beam optical trap^20^. Accordingly, multiple RBM peaks due to a large number of tubes were observed from the trap volume^20^,^21^. Here, we typically observed one sharp RBM peak for one trapped tube. It was found that the frequency of the RBM of an isolated SWCNT can be experimentally determined by ω_RBM_ = 248 cm^−1^/d_t_, where d_t_ is the diameter of the nanotube^39^. ω_RBM_ at 266 cm^−1^ of the trapped nanotube in Fig. 1d corresponds to a diameter of 0.93 nm. Due to the resonance nature, different RBM might be observed when the exciting wavelength is changed^39^. We found that some individual tubes can be trapped both in single-beam OT and the SWOT, yet their trapping in the SWOT was more stable such that the Brownian motion of the trapped tube extended in a less region. We also found that many individual tubes cannot be trapped in single-beam OT, but they can be stably trapped in the SWOT. To observe this phenomenon, an individual tube was moved into the focused laser beam by steering the translation stage of the microscope and it will be pushed upward if it cannot be trapped by single-beam optical trap. The tube will continue to be pushed towards to the reflecting surface until it is trapped in a SWOT by adjusting the focus position of the objective upward.

**Figure 2:**
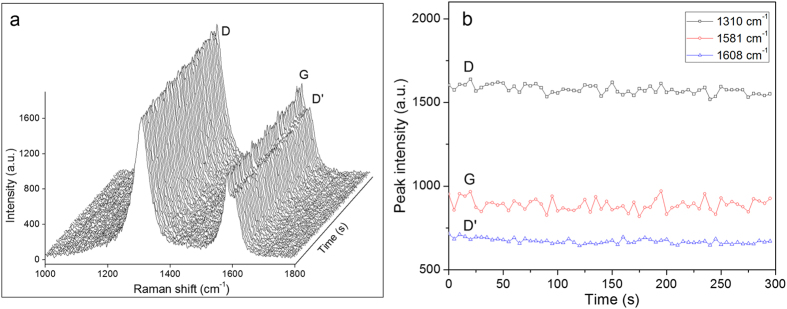
(**a**) Time-lapse Raman spectra of an individual optically trapped SWCNT in a SWOT in solution for 5 min. The SWCNT has a length of 2.1 μm. The ensemble of 60 spectra was acquired at intervals of 5 s with the laser power of 5 mW, over a period of 5 min. (**b**) The dynamics over time of the signal intensities of the three peaks (D, G, and D′).

To demonstrate stable trapping of an individual SWCNT in the SWOT, we recorded time-lapse Raman spectra of an optically trapped individual SWCNT for 5 min acquired at intervals of 5 s with the laser power of 5 mW. Figure 2a shows the time-lapse Raman spectra and Fig. 2b shows the dynamics over time of the signal intensities of the three peaks (D, G, and D′). In contrast to the previous observation of increasing concentration of SWCNTs in the confocal volume of a single-beam optical trap^20^, both the Raman spectra and peak intensities of the trapped SWCNT were unchanged over a period of 5 min or longer with a laser power of 5 mW (Fig. 2a,b), suggesting stable trapping of an individual SWCNT in SWOT.

We found that some individual graphene flasks and MWCNT can be trapped in SWOT and their Raman spectra can be measured (Fig. 1d, Figure S1a). The laser power for trapping and analyzing an individual MWCNT was decreased to 1.5 mW to avoid the formation of bubbles by laser heating, since the absorption of MWCNTs is much stronger than that of SWCNTs and this larger absorption makes the trapping of MWCNTs be relatively difficult.

**Figure 3:**
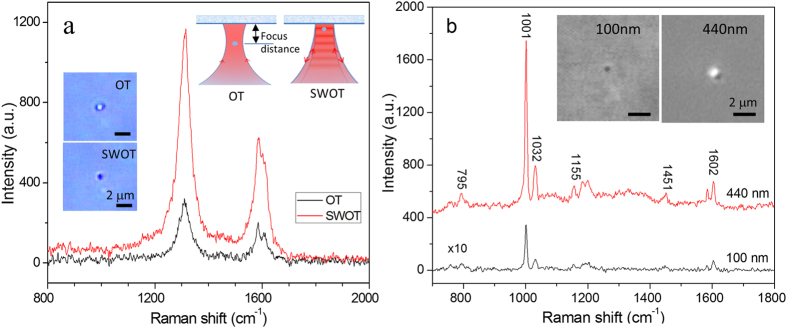
(**a**) Raman spectra of an optically trapped SWCNT when the focus distance of the trapping beam is >5 μm away from the reflecting mirror, corresponding to single-beam optical tweezers (OT), or the trapping beam is focused onto the reflecting beam, corresponding to SWOT. Insets are the images of the same SWCNT when it is in OT or in SWOT. (**b**) Raman spectra of single trapped polystyrene beads of 100 nm and 440 nm in diameter. Inset shows the bright-field image for the trapped 100-nm bead (see Supplementary Movie 2) and DIC image for the trapped 440-nm bead with a scale bar of 2 μm. The laser power is 5 mW and the acquisition time is 20 s and 2 s for 100-nm bead and 440-nm bead, respectively.

To compare the Raman spectra of a trapped particle in a single-beam Raman tweezers and a SWRT, we captured a single nanoparticle in a single-beam OT when the focus distance of the trapping beam was adjusted far away from the reflecting mirror, and then axially manipulated the trapped particle to the SWOT. Figure 3a shows Raman spectra of an optically trapped SWCNT with a 2.1-μm length when the focus distance of the trapping beam was moved from >5 μm away from the reflecting mirror (corresponding to OT) onto the reflecting beam (corresponding to SWOT). The D peak intensity of the trapped tube in SWOT was 3.82 ± 0.12 times higher than that in OT, indicating that Raman signals in a SWRT was increased by a factor close to 4-fold. Insets are the images of the trapped SWCNT when it is in OT or SWOT. Profile analysis of the image pixel intensity by ImageJ indicated that the lateral image size of the trapped tube was 0.34 μm in SWOT (close to the diffraction limit), while it was 1.30 μm in traditional OT, suggesting the trapping with a SWOT is more stable so that the tube looks darker and smaller.

### Enhancement of Raman scattering signals of optically trapped polystyrene beads

For a quantitative comparison, we also studied the Raman spectra of individual polystyrene beads of 2-μm in diameter which can be easily trapped and manipulated by a single-beam OT, while the focus of the trapping beam was adjusted from far away from the reflecting mirror to focusing on the reflecting mirror. Figure S2a shows Raman spectra of a polystyrene bead trapped in an OT (focusing 5-μm away from the reflecting mirror) or in a SWOT. The insert shows the normalized peak intensities of the 1001 cm^−1^ band as the function of the focus distance of the trapping laser from the reflecting mirror, which was precisely adjusted with a piezoelectric focusing system (MIPOS 100PL, Piezosystem Jena Inc). The data indicated that the enhancement factor of the Raman signals was 3.24 ± 0.51 in the SWOT, which was averaged over ten measured beads. Since the diameter of 2-μm polystyrene bead is greater than the distance between two antinodes (~0.3 μm), the trapped bead covers several nodes and antinodes of the standing-wave. The average laser intensity for the bead in SWOT is twice the intensity of the incident beam and thus the Raman signal is increased by a factor of ~4-fold.

We also tried to use 100-nm polystyrene beads (d < λ/2n) to test if the Raman enhancement factor is close to 8-fold. However, individual 100-nm polystyrene beads cannot be steadily trapped in single-beam OT for Raman analysis even with a trapping power increased to 50 mW, although they can be easily trapped in a SWOT with an incident power of 5 mW. Alternatively, we compared Raman spectra of an individual 100-nm polystyrene bead and an individual 440-nm polystyrene bead, which were optically trapped in SWOT respectively. The 440-nm bead had a size larger λ/2n and was found to have an enhancement factor of ~3.6 for Raman spectra in SWOT. Figure 3b shows Raman spectra of an individual 100-nm polystyrene bead and an individual 440-nm polystyrene bead. The laser power was the same as 5 mW and the acquisition time was 20 s and 2 s for the 100 nm-bead and the 440 nm-bead, respectively. The ratio of their Raman peaks at 1001 cm^−1^ was 2.62 × 10^−2^ after normalizing the acquisition time, which should be proportional to their volume ratio and Raman enhancement factors. Considering that the volume ratio of the 100-nm bead and the 440 nm-bead as 1.17 × 10^−2^, we estimated that the enhancement factor for the 100-nm bead as 8.0 ± 0.3.

For transparent polystyrene beads with a diameter of ~100 nm, they are difficult to be trapped steadily and characterized using single-beam Raman tweezers with a relatively low power of 5–20 mW. Since the gradient force acting on these small particles is significantly small to compete with Brownian motion, the lifetime of these nanoparticles in single-beam OT is too short to measure their Raman spectra^19^. However, these nanoparticles can be easily trapped and manipulated using a SWOT. Supplementary Video 2 (in Supplementary Information) shows optical trapping and manipulation of an individual 100 nm polystyrene bead with the 5-mW trapping laser beam. When the laser beam was turned off, the polystyrene bead moved rapidly due to Brownian motion. When the laser beam was turned on, the polystyrene bead was pushed towards to the reflecting mirror and trapped steadily in a SWOT. The trapped particle can be then manipulated across the sample well by moving the translation stage or steering the incident laser beam transversely. We found that the trapped 100-nm particle can stay in the SWOT stably for more than 10 min for Raman spectroscopy measurements if no other particles were pulled into the trap and collided with it. Figure 3b shows the Raman spectrum of an individual 100-nm polystyrene bead optically trapped in a SWOT. We also measured the Raman spectra of individual nano-sized polystyrene beads of 200 nm, 300 nm, and 440 nm in diameter (Bangs Laboratories Inc.) trapped in a SWOT and found that their relative intensities of the 1001 cm^−1^ peak fitted well to the cubic of the diameter (Figure S2b).

### Raman spectroscopy of individual optically trapped high-refractive titanium dioxide and silicon nanoparticles

Single-beam Raman tweezers are limited to analyze many kinds of nanoparticles dispersed in liquid because of their small sizes and high refractive index such as semiconductor nanocrystals of silicon. For nano-particles with a high-refractive index such as titanium dioxide (n = 2.52) and silicon (n = 3.71), the lateral optical force acting on high-refractive index particles by a single beam OT is larger than that acting on low-refractive index particles^38^. However, the scattering force along the laser propagating direction becomes much larger than the axial gradient force and, therefore, it is hard to trap steadily high-refractive index particles with a Gaussian beam optical trap^40^. It is possible to trap high-refractive index particles with two counter-propagating beams or a SWOT because the two counter-scattering forces will be balanced. In this experiment, we showed that individual titanium dioxide or silicon nanocrystals can be steadily trapped and manipulated in a SWOT, and then characterized by their Raman spectroscopy (see Figure S1b). The band at 642 cm^−1^ in TiO_2_ particle is assigned to the E_g_ mode, the band at 401 cm^−1^ to the B_1g_ mode, and the band at 518 cm^−1^ to a doublet of the A_1g_ and B_1g_ modes^41^. The characteristic band at ~519 cm^−1^ in Si nanocrystals is assigned to crystalline phase of silicon^42^.

We also showed that single aggregates of 50-nm carbon particles or graphite particles can be trapped in a SWOT and characterized by their Raman spectroscopy (see Figure S1a). We found that only small size carbon clusters (typically smaller than 300 nm) can be trapped steadily with a SWOT and the trapping power had to be below 5 mW, because the heat generated by carbon clusters may disturb the stability of the SWOT.

### Optical trapping and surface-enhanced Raman spectroscopy (SERS) of cluster of metal nanoparticles

**Figure 4:**
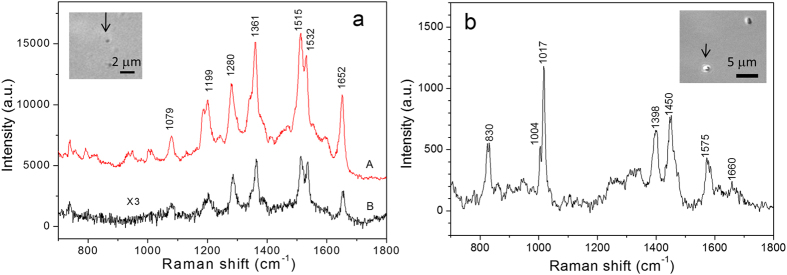
(**a**) Raman spectrum of a cluster of 80-nm gold particles in SWOT dispersed in a water solution containing 2.5 μM of Rhodamine B (curve A), in comparison to the spectrum of 100 mM bulk Rhodamine B medium (curve B). The insert shows the bright-field image of the trapped gold particle. The laser power was 2 mW and 5 mW for A and B, respectively, and the integration time was 1 s. (**b**) Raman spectrum of a single trapped *B. cereus* spore in SWOT. The insert shows the DIC images of the trapped spore. The laser power was 5 mW and the integration time was 10 s.

Previous studies showed that SERS-active metal nanoparticles can be optically trapped by a single-beam OT for high-sensitivity label-free identification of molecular species^24^,^25^,^26^. Optical forces have been used to bring two Ag nanoparticles into contact to create a SERS-active dimer that is capable of strongly enhancing the Raman signal of thiophenol molecules (10 μM) contained in a liquid solution^24^. In general, metal particles are difficult to trap by single-beam OT due to their high reflectivity, which causes the strong scattering force to push the particles along the optical axis^17^. Optical trapping of metal nanoparticles is allowed if the laser frequency is far enough below the localized surface plasmon (LSP) resonance frequency with a high power^24^ or plasmonic nature of metal nanoparticles enhances the gradient force^18^. In this experiment, we showed that metal nanoparticles are more easily and stably trapped in a SWOT with a low laser power because the strong scattering forces are balanced in a standing-wave. It is thus more easily to create nanoparticles cluster that will strongly enhanced Raman signal of analyte molecules contained in the liquid solution. The 80-nm in diameter gold spherical particles with LSP resonance wavelength at 544 nm (Lot No. ARS1292, Ted Pella, Inc) were dispersed in distilled water and Rhodamine B molecules was used with a final concentration of 2.5 μM as our probe molecule. The power of the incoming laser was reduced to 2 mW to reduce the heating effect. We found that most individual 80-nm gold particles cannot be trapped in single-beam OT with this low power, but they were pushed towards to the reflecting mirror and stably trapped in the SWOT. Figure 4a shows the Raman spectrum of a small cluster of 80-nm gold particles in SWOT dispersed in a water solution containing 2.5 μM Rhodamine B acquired with 2-mW laser power, in contrast to the Raman spectrum of a reference Rhodamine B solution (100 mM) acquired with 5-mW laser power and an integration time of 1 s. The insert shows the bright-field image of a trapped aggregate of gold particle which is smaller than 300 nm. It was known that gold aggregates usually yield a strong SERS response compared to isolated nanoparticles, because the former enables additional strong field enhancement in the gap regions between the particles. Comparing the peak intensities of these spectra, we estimated that the enhancement factor for SERS was >6.0 × 10^5^. The SERS enhancement factor was calculated by EF = [(I_SERS_ /c_SERS_)/(I_R_ /c_R_)]*(P_R_/P_SERS_), where I_SERS_, c_SERS_, and P_SERS_ are the Raman intensity of the 1515 peak, the analyte concentration, and the laser excitation power for the SERS sample, I_R_, c_R_, and P_R_ the Raman intensity of the 1515 peak, the analyte concentration, and the laser excitation power for the reference sample. This enhancement factor was well under-estimated because the molecule number nearby the surface of the trapped gold particle is much less than that in the confocal volume in bulk sample. We also noticed that if the laser power was increased to >5 mW, the SERS signals of Rhdomine B adsorbed on the surface of gold particles were hard to observe, and the trapped gold cluster quickly adhered to the reflecting mirror surface (within a few seconds after the cluster entered the laser beam). We contributed this phenomenon as the heating effect due to the plasmon-enhanced absorption of gold nanoparticles, which dramatically disturbed the SWOT stability.

### Optical trapping and analysis of biological cells

We also show that the SWRT can be used to trap and characterize biological particles. Figure 4b is the Raman spectrum of a single optically trapped *Bacillus cereus* spore in SWOT. The insert shows the DIC images of the trapped spore. The *B. cereus* bacterial spore has a size of about 1.5 μm, covering several nodes and antinodes of the standing wave. The Raman signal intensity of the trapped spore in SWOT is about four-fold of that measured by a single-beam Raman tweezers with the same incident power. The spectrum contains several CaDPA bands at 1017, 1398, 1450, and 1575 cm^−1^, suggesting that this trapped bacterium was in a dormant state^12^,^13^.

## Conclusions

In summary, we have developed a standing-wave Raman tweezers for stable trapping and label-free characterization of nanostructures by combining confocal Raman spectroscopy and standing-wave optical traps. The standing-wave Raman tweezers is more stable and sensitive in measuring nanoparticles in liquid with 4–8 fold increase in the Raman signals and it can be used to trap and analyze many nanoparticles that cannot be measured with single-beam Raman tweezers. Consequently, the SWRT can be utilized to manipulate individual nanoparticles of different materials including SWCNTs, graphene flakes, polystyrene beads (100 nm), gold particles (80 nm), high-refractive titanium dioxide, and silicon nanoparticles and analyze them by Raman fingerprints with a low laser power of a few mW. The standing-wave Raman tweezers can also be used to capture individual SERS-active metal nanoparticles and biological particles, a methodology that allows in principle for highly-sensitive label-free analysis of molecules. The SWRT could be applied for sorting specific SWCNTs and for assembly and characterization of nanodevices.

## Additional Information

**How to cite this article**: Wu, M.- *et al*. Stable optical trapping and sensitive characterization of nanostructures using standing-wave Raman tweezers. *Sci. Rep.* **7**, 42930; doi: 10.1038/srep42930 (2017).

**Publisher's note:** Springer Nature remains neutral with regard to jurisdictional claims in published maps and institutional affiliations.

## Supplementary Material

Supplementary Video 1

Supplementary Video 2

Supplemental Materials
