# Supplementary material for: Stable optical trapping and sensitive characterization of nanostructures using standing-wave Raman tweezers
Source: Sci Rep. 2017 Feb 17;7:42930. doi: 10.1038/srep42930 (PMC5314326; doi:10.1038/srep42930)
Supplement: Supplemental Materials [file srep42930-s3.pdf]

Supplementary Information

# Stable optical trapping and sensitive characterization of nanostructures using standing-wave Raman tweezers

*Mu-ying Wu[1], Dong-xiong Ling[1], Lin Ling[2], William Li[2], and Yong-qing Li[1,2*]*

[1]School of Electronic Engineering, Dongguang University of Technology, Dongguan, Guangdong, P.R. China

[2]Department of Physics, East Carolina University, Greenville, North Carolina 27858-4353, USA

*Correspondence email: liy@ecu.edu

**Additional information.** A movie (Supplementary Video1) showing optical trapping and manipulation of an individual single-walled carbon nanotube (SWCNT) using a standing-wave Raman tweezers with the incoming laser beam on or off; a movie (Supplementary Video2) showing optical trapping and manipulation of an individual 100 nm polystyrene bead with the incoming laser beam on or off; the Raman spectra (Figure S1) of a trapped single-walled carbon nanotube, multi-walled carbon nanotube, an aggregate of 50-nm carbon particles, a graphite particle, as well as single trapped $TiO_2$ and Si nano-particles; and the Raman spectra (Figure S2) of a polystyrene bead in an OT or in a SWOT are available in Supplementary Information.

## Figure S1

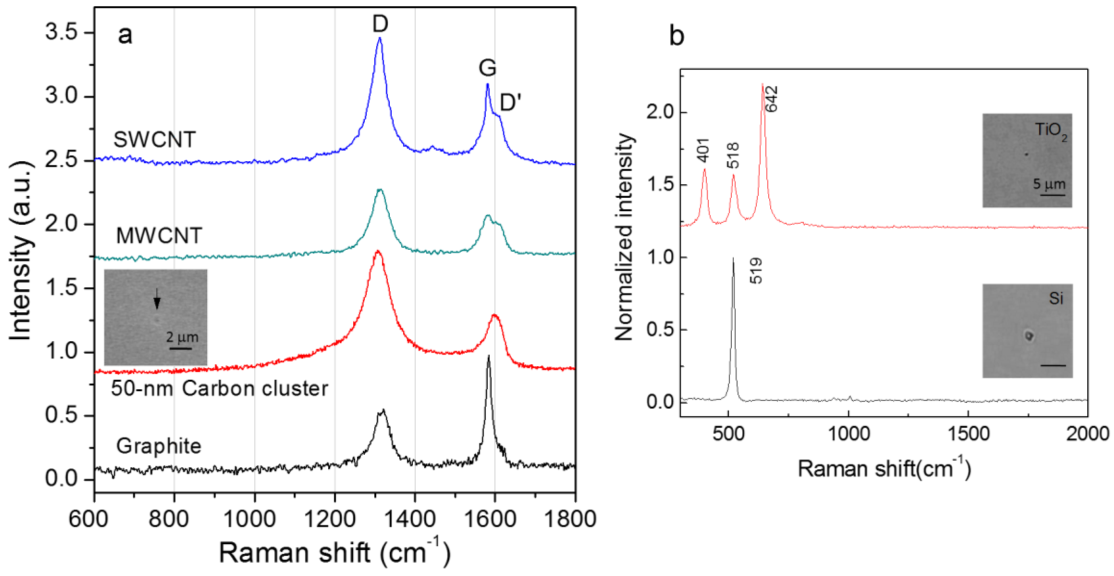


**Figure S1.** (a) Raman spectra of a trapped SWCNT, MWCNT, an aggregate of 50-nm carbon particles and a graphite particle. The laser power is 5 mW at 780 nm and the acquisition time is 1s. The inset is the image of the aggregate of 50-nm carbon particles with the scale bar of 2 µm. (b) Raman spectra of single trapped $TiO_2$ and Si nano-particles. The laser power is 10 mW and 25 mW with an acquisition time of 1 s for $TiO_2$ and Si particle, respectively. The scale bar is 5 µm.

**Figure S2**

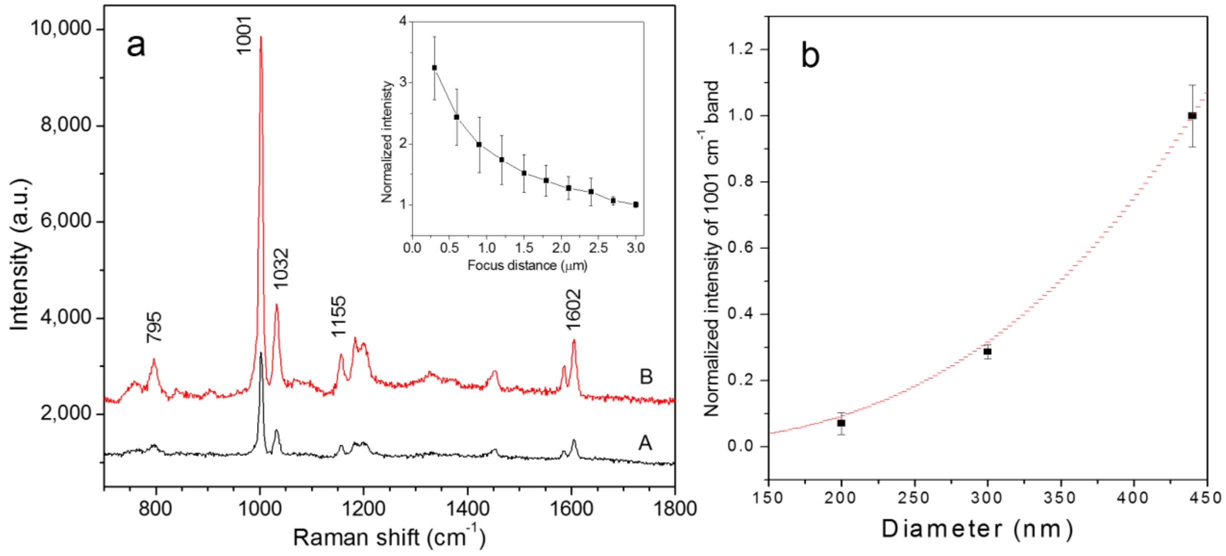


**Figure S2**. (a) Raman spectra of a 2-μm polystyrene bead in an OT (curve A) or in a SWOT (curve B). The insert shows the normalized peak intensities of the 1001 $cm^{-1}$ band as the function of the focus distance of the trapping laser from the reflecting mirror. (b) Relative intensity of the 1001 $cm^{-1}$ band of nano-sized polystyrene beads of 200nm, 300nm, and 440 nm in diameter. The dotted line is the value of the cubic of the diameter.

**Supplementary Video 1.** A movie showing optical trapping and manipulation of an individual single-walled carbon nanotube (SWCNT) using a standing-wave Raman tweezers with the incoming laser beam on or off.

**Supplementary Video 2.** A movie showing optical trapping and manipulation of an individual 100 nm polystyrene sphere with the incoming laser beam on or off.
